# Design and 4D Printing of Cross-Folded Origami Structures: A Preliminary Investigation

**DOI:** 10.3390/ma11030376

**Published:** 2018-03-03

**Authors:** Joanne Ee Mei Teoh, Jia An, Xiaofan Feng, Yue Zhao, Chee Kai Chua, Yong Liu

**Affiliations:** Singapore Centre for 3D Printing, School of Mechanical & Aerospace Engineering, Nanyang Technological University, Singapore 639798, Singapore; anjia@ntu.edu.sg (J.A.); Feng0080@e.ntu.edu.sg (X.F.); zhao0194@e.ntu.edu.s (Y.Z.); MCKCHUA@ntu.edu.sg (C.K.C.); yliu.email@yahoo.com (Y.L.)

**Keywords:** 4D printing, smart structures, shape memory polymer, polyjet, additive manufacturing, cross-folding

## Abstract

In 4D printing research, different types of complex structure folding and unfolding have been investigated. However, research on cross-folding of origami structures (defined as a folding structure with at least two overlapping folds) has not been reported. This research focuses on the investigation of cross-folding structures using multi-material components along different axes and different horizontal hinge thickness with single homogeneous material. Tensile tests were conducted to determine the impact of multi-material components and horizontal hinge thickness. In the case of multi-material structures, the hybrid material composition has a significant impact on the overall maximum strain and Young’s modulus properties. In the case of single material structures, the shape recovery speed is inversely proportional to the horizontal hinge thickness, while the flexural or bending strength is proportional to the horizontal hinge thickness. A hinge with a thickness of 0.5 mm could be folded three times prior to fracture whilst a hinge with a thickness of 0.3 mm could be folded only once prior to fracture. A hinge with a thickness of 0.1 mm could not even be folded without cracking. The introduction of a physical hole in the center of the folding/unfolding line provided stress relief and prevented fracture. A complex flower petal shape was used to successfully demonstrate the implementation of overlapping and non-overlapping folding lines using both single material segments and multi-material segments. Design guidelines for establishing cross-folding structures using multi-material components along different axes and different horizontal hinge thicknesses with single or homogeneous material were established. These guidelines can be used to design and implement complex origami structures with overlapping and non-overlapping folding lines. Combined overlapping folding structures could be implemented and allocating specific hole locations in the overall designs could be further explored. In addition, creating a more precise prediction by investigating sets of in between hinge thicknesses and comparing the folding times before fracture, will be the subject of future work.

## 1. Introduction

Although origami is a type of traditional art where a piece of flat paper is folded into a 3D object, the notion of origami is widely explored nowadays to provide innovative solutions to the problems of compacting large objects into a small volume of space. For instance, applications of origami can be found in the airbags for automobiles, cartons and shopping bags, and photovoltaic solar cells with shape changing ability. However, the packing process is often challenging and may lead to an increase in the infrastructure cost since new equipment may be required if any changes in folding design are needed. Therefore, the idea of active origami is intriguing as it can help to reduce the investment needed for the folding equipment. 

Active origami is defined as a design to create an origami object that has the ability to self-fold or self-unfold [1]. The structure is first printed using a 3D printer and then is driven by an external stimulus to reprogram its shape or properties over time. 4D printing is the printing of a dynamic 3D structure that is capable of changing its shape over time [2,3]. In order to do that, smart materials are 4D printed by using fused deposition modelling of single or double layer print [4,5], 3D projection and laser stereolithography (SLA) [6], digital light processing printing (DLP) [7], PolyJet technology [8] or Selective Laser Melting (SLM) [9]. For example recently, there has been research performed on the design of origami by using SMPs (Shape Memory Polymers), light activated polymers [10], and shape memory alloys [11,12]. 

Moreover, with the recent improvement in the multi-material printing technology of additive manufacturing [13], multiple materials can be positioned precisely in terms of micrometer scales without any constraints on the geometric complexity of the manufactured 3D components [3,14]. Reversibility of SMP has also been reported [15]. In addition, this advancement of technology allows complicated 3D components with non-uniform material distributions and multifunctional performance to be produced as well. 

Additional research on printed active composites has also been carried out to have a better understanding of the impact of fiber volume fraction and fiber orientation (loading direction) on complex shape programming and recovery. The maximum bending angle of hinges was determined to decrease monotonically with respect to the inclined angle between the fiber and the loading direction which affects the axial strain of embedded fibers. This physical response characteristic parameter was implemented to achieve shape programming and recovery in complex structures [16]. 

Another research area to concentrate on is to understand and achieve controlled sequential folding or shape recovery of the active origami [17,18,19]. As the design of the origami becomes more complex, folding of the active origami parts at the same instant might cause the different folding parts to interfere with each other before the folding process is complete. Moreover, very little research has been done on the study of controlled sequential shape recovery of SMPs or *active origami*. 

**Figure 1:**
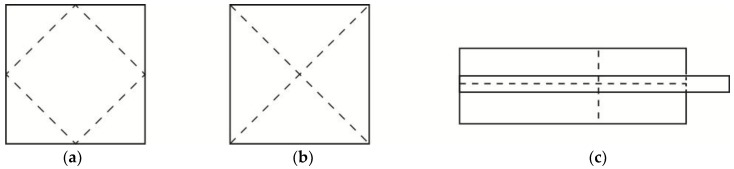
Multifolding. (**a**) Non-overlapping folding lines; (**b**) Overlapping folding lines, and (**c**) combination of overlapping and non-overlapping folding lines.

However, all the current 4D printed origami structures fold or unfold in a similar way without overlapping of the folding lines. Folding and unfolding of structures are essential in 4D printing. Usually, structures fold or unfold along a certain axis. A folding line is defined as a segment of the folding axis on the structure. In a simple structure such as a hinge, there is only one folding line. In a structure involving multifolding, there are multiple folding lines. However, these folding lines may or may not overlap or cross each other (see Figure 1). Figure 1a shows a typical configuration of folding lines of current 4D printed origami structures. Crossfolding is defined as a multifolding in which at least two folding lines within the same plane overlapseach other (Figure 1b). Figure 1c shows a design with a combination of overlapping and non-overlapping folding lines.

Crossfolding allows the deformation of a structure to be doubled i.e., saving 50% of the space in a single fold. In this research, four different materials (VeroWhitePlus, DM8510, DM8520, and DM8530) and three different axes (X, Y, and Z) are selected to explore the feasibility of printing and programming crossfolded smart structures as well as to characterize crossfolded structures. Mechanical properties of single material and multimaterials are first analyzed and then crossfolded single material structures and multimaterial structures are designed to achieve higher complexity. The rule of mixture for multi-materials is used to predict the overall Young’s Modulus of the structure based on the moduli of the individual constituents and their volume fractions. Single material specimens are used to determine the shape recovery characteristics for using the microforce test. A hole was designed at the crossing point to study its effect on stress concentration.

## 2. Materials and Methods 

The materials used in this research are based on two basic proprietary materials provided by Stratasys (Eden Prairie, MN, USA), namely TangoBlackPlus (TB) and VeroWhitePlus (VW). Compared to TB, which is rubbery and has a great extent of elongation, VW is a rigid and opaque material that has high Young’s modulus, great tensile strength, and 10–25% of elongation before break. TB liquid resin comprises urethane acrylate oligomer, exo-1,7,7-trimethylbicyclo hept-2-yl acrylate, methacrylate oligomer, polyurethane resin, and photoinitiator. VW liquid resin consists of isobornyl acrylate, acrylic monomer, urethane acrylate, epoxy acrylate, acrylic monomer, acrylic oligomer, and photoinitiator. By mixing these two materials, new materials possessing different glass transition temperatures (T_g_) can be obtained. In this research, four materials, VW and three mixtures from VW and TB (DM8510, DM8520, and DM8530) were used. All three mixtures are Stratasys’ proprietary digital materials (DM), with VW as the primary component and TB the secondary component. The percentage of TB in each has not been released by Stratasys, but the order is known as follows: DM8510 < DM8520 < DM8530 (highest percent of TB) [20,21].

### 2.1. Preparation of Single Material Specimens and Tensile Test

All specimens used in this Microforce mechanical testing were printed using Objet 500 Connex 3 Polyjet printer (Stratasys, Eden Prairie, MN, USA). The four materials used are VeroWhitePlus, DM8510, DM8520, and DM8530. Five specimens were 3D printed for each material. Among the four materials, DM 8510, DM 8520, and DM 8530 are digital materials that are combinations of two materials. They all have VeroWhitePlus as primary material and TangoBlackPlus as secondary material. The difference is that the percentage of TangoBlackPlus material increases from DM 8510 to DM 8530 (refer to Appendix B and Appendix C).

**Figure 2:**
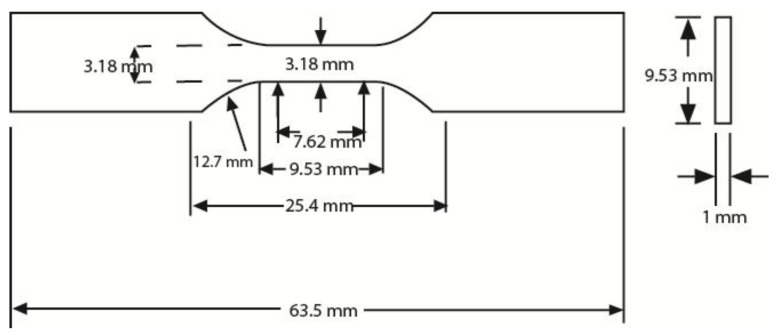
Type V: specimen dimensions.

According to the ASTM D638 standard, for rigid and semi-rigid plastics, type I and type II specimens are to be used with materials having a thickness of 7 mm [22]. Type III specimen must be used for material having a thickness greater than 7 mm, while type IV specimen is to be used when there is a need to compare materials in different rigid cases [22]. Type V specimen is to be used for material having a thickness of 4 mm or less [22]. Thus, for the specimen used in this tensile test, ASTM D638 type V was chosen and is shown in Figure 2. 

The MTS Criterion Model 43 machine from MTS Systems Corporation in the United States was used for this microforce test. The specimen was clamped with alignment and held without any slippage to the grips. The load was measured using a load cell and the displacement of the crosshead was set to be 3.5 mm. The heating chamber of the machine was used to heat up the specimen to 70 °C and maintain the temperature. 

A thermocouple was used to monitor the surface temperature of the specimen for 10 min. This was to ensure complete heat transfer across the specimen.

A temperature-controlling water tank was used for reheating the specimen after measuring the elongation.

### 2.2. Calculation of Shape Recovery for Single Material Specimens

**Figure 3:**
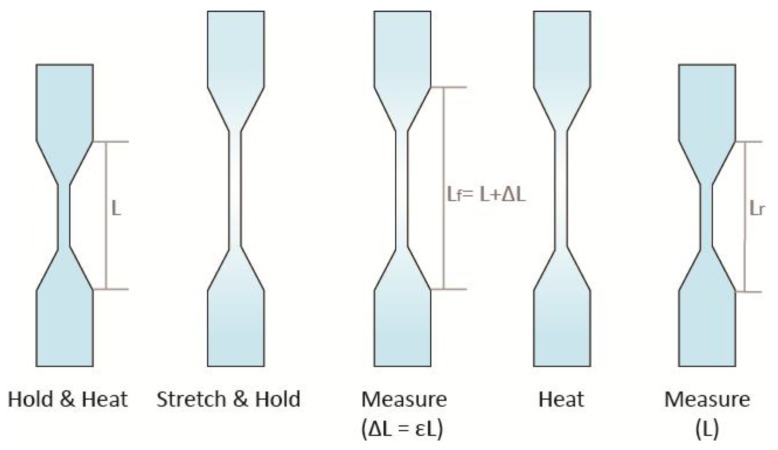
Shape recovery property measurements.

Figure 3 shows the steps of the shape setting method which were used to determine the shape recovery characteristics for single material specimens.

- For every specimen of four different materials, the specimen was held using tensile grips at a room temperature of 25 °C.
- The displacement of the crosshead was set to 3.5 mm and the load calibration was applied.
- The chamber was heated to an equilibrium temperature of 70 °C and tensile testing was performed.
- After the tensile test was completed, the chamber temperature was reduced to 25 °C with the specimen still being held by tensile grips and the change in length was measured.
- The specimen was placed in a hot water batch of 70 °C for shape recovery and the change in length was measured again.

After the final gauge length L_f_ was measured, the percent elongation was calculated using the formula:

%El = (L_f_ − L_o_)/L_o_) × 100% (1)

Similarly, the gauge length L_r_ after recovery by reheating was measured and the percent recovery was calculated using the formula:

%R = (1 − (L_r_ − L_o_)/L_o_) × 100% (2)

The original gauge length L_o_ is 7.62 mm.

Percentage of recovery rate is defined by the following:

%R_r_ = (1 − (L_r_ − L)/ΔL) (3)

### 2.3. Design and Crossfolding of Single Material Smart Structures

**Figure 4:**
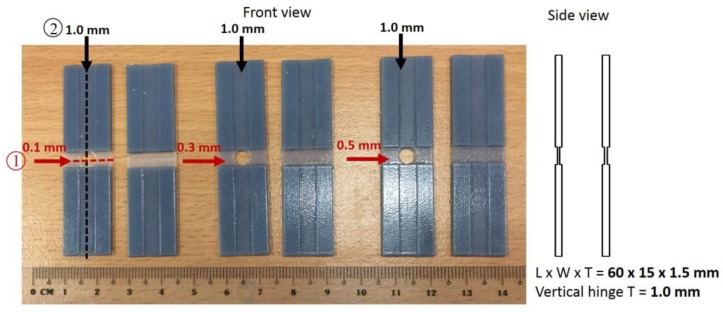
Structures with crossed folding lines of different thicknesses.

Based on the tensile test results, DM8530 material had near 100% recovery. Therefore it was selected to study the influence of hinge thickness in the multifolding process. A series of tests were conducted using DM8530 material. All the selected four materials display good and similar shape recovery properties (See Figure 14 later). Therefore we used DM8530 as a representative of the four for cross-foldability study. We expected the other three materials to perform the same way as DM8530 when 4D printed. There were 12 specimens of Length × Width × Thickness = (60 × 15 × 1.5) mm. Each specimen was designed with two crossed folding lines as shown in Figure 4, For folding line 1, the thickness (note: not the width of the channel) was 0.1 mm, 0.3 mm, and 0.5 mm thickness and for folding line 2, the thickness was a constant of 1.0 mm. There were two samples with hole and two samples without hole for each thickness. A hole was designed at the crossing point to eliminate the overlapping crossfolding region. 

Results for design and crossfolding of single material smart structures are described in Section 3. 

### 2.4. Preparation of Multimaterial Specimens for Tensile Test

**Figure 5:**
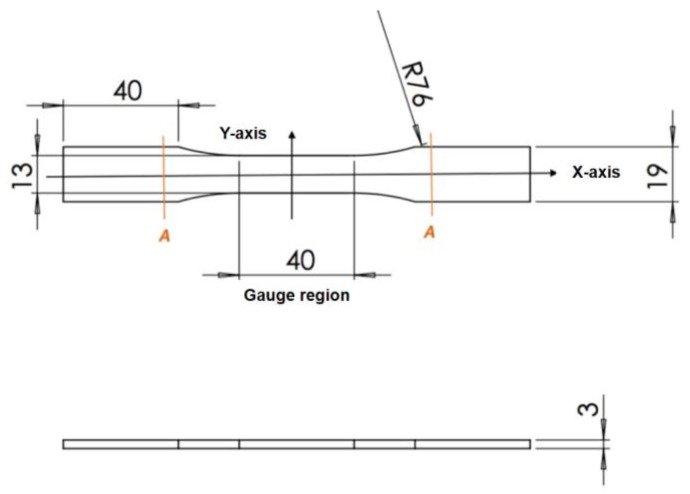
Specimen dimensions.

According to the ASTM D638 standard, the specimen dimensions are given in Figure 5.

Different material combination information is given in Table 1. In each combination, two materials were combined symmetrically along the X-axis, Y-axis, and Z-axis (here the Z-axis refers to the axis normal to the XY plane). Table 2 shows the specimen material composition.

ASTM D638 standard tensile tests were performed on all specimens at a room temperature of 25 °C.

The testing speed was given as 3 mm/min to comply with ASTM D638, which gave a strain rate of $1.25\times 10^{-3\;}s^{-1}$.

### 2.5. Rule of Mixtures for Multimaterial Specimens 

**Figure 6:**
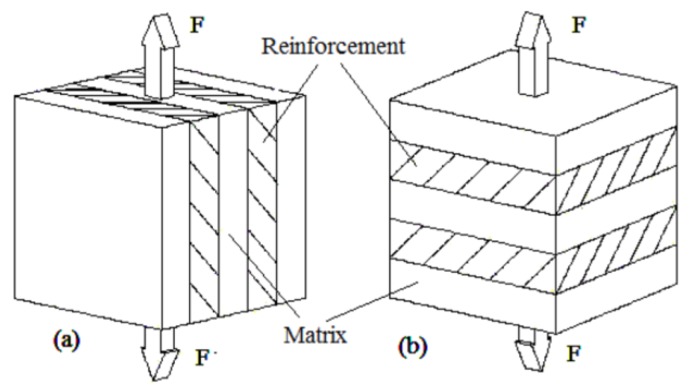
(**a**) Voigt iso-strain model; (**b**) Reuss iso-stress model.

The rule of mixtures is a mathematical expression which describes some properties of the composite in terms of the properties, quantity, and arrangement of their constituents [23]. It can be used to predict the overall Young’s Modulus of the mixture based on the moduli of the individual constituents and their volume fractions [24]. The properties, i.e., Young’s Modulus, are proportional to their volume fractions of the components and lie between the pure component values [25]. In this research, different multi-materials can be approximated as composites to obtain predicted Young’s Modulus. Two established methods were practiced to calculate the Young’s Modulus of the mixtures, namely iso-strain and iso-stress. The two simple models widely used are the Voigt iso-strain model and the Reuss iso-stress model (Figure 6) [26].

**Figure 7:**
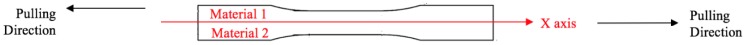
Modelling X-axis combination as iso-strain.

**Figure 8:**
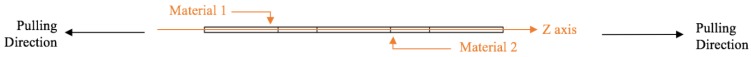
Modelling Z-axis combination as iso-strain.

As the name implies, iso-strain requires the composite components to undergo the same deformation strain, whereby the load direction is the same as the fiber direction [27]. In our case, the whole specimen is the composite. Material 1 can be approximately treated as a matrix while material 2 can be approximately treated as a fiber. Iso-strain can be applied to the multi-material printing orientation of the X-axis and Z-axis (XY plane) as illustrated below in Figure 7 and Figure 8. Both materials undergo the same deformation strain.

For the iso-strain condition, the Young’s Modulus of the composite can be calculated by Equation (4) [28]:

(4) $$E_{c}=E_{m}V_{m}+E_{p}V_{p}$$

In our case, the equation is modified to the following Equation (5):

(5) $$E_{s}=E_{m1}V_{m1}+E_{m2}V_{m2}$$

Young’s Modulus for material 1 and 2 are known separately from previous tests. Volume fractions are 50% for matrix (material 1) and fiber (material 2). Since $V_{m1}=V_{m2}=$ 50%, the equation can be rewritten in the following form:

(6) $$E_{s}=V_{m}\left(E_{m1}+E_{m2}\right)$$

Hence, the choice of material 1 or material 2 as matrix or fiber will not affect the result. Young’s Modulus for the specimen can then be calculated.

**Figure 9:**
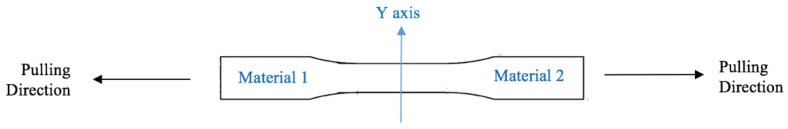
Modelling Y-axis as iso-stress.

On the other hand, iso-stress refers to the condition whereby load is applied normal to the orientation of the fibers [27]. The normal stresses for matrix and fibers are the same. This condition can be applied to the multi-material printing orientation of the Y-axis as illustrated in Figure 9. Since both materials have the same cross-section, upon application of the load, they will have the same normal stress according to Equation (7):

(7) $$\sigma =\frac{F}{A}$$

where *F* is the force applied and *A* is the cross-sectional area [29].

For the iso-stress condition, Young’s Modulus of the composite can be calculated by Equation (8) [28]:

(8) $$E_{c}=\frac{E_{m}V_{f}+E_{f}V_{m}}{E_{m}E_{f}}$$

In our case, the equation is modified to the following Equation (9):

(9) $$E_{c}=\frac{E_{m1}V_{m2}+E_{m2}V_{m1}}{E_{m1}E_{m2}}$$

Similarly, Young’s Modulus for material 1 and 2, known separately from previous tests and volume fractions, are both 50%:

(10) $$E_{c}=\frac{E_{m1}+E_{m2}}{E_{m1}E_{m2}}V_{m}$$

Hence again, it does not matter whether material 1 or material 2 is treated as a matrix or a fiber.

### 2.6. Design and Crossfolding of Multimaterial Smart Structures

**Figure 10:**
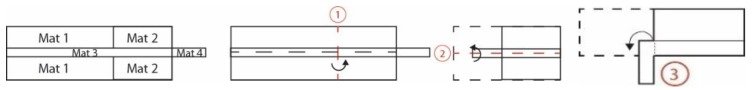
Sequence of crossfolding for method 1.

**Figure 11:**
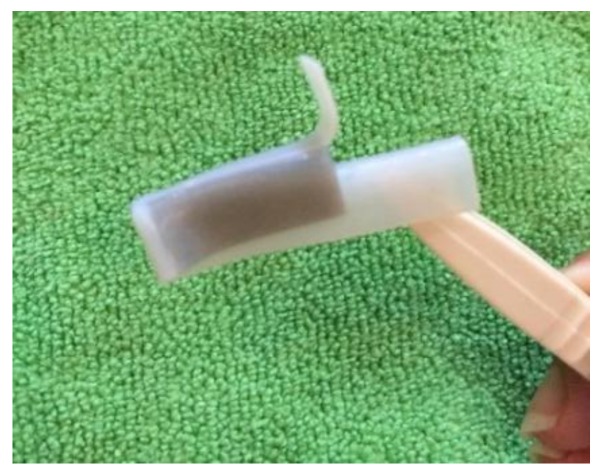
Crossfolding of final structure of method 1.

**Figure 12:**
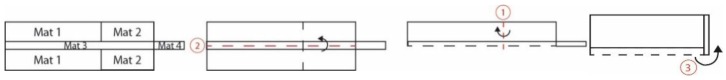
Sequence of crossfolding for method 2.

There are two types of folding: folding at not more than 90° and folding at more than 90°. Crossfolding requires the first folding angle to be close to 180° before the second fold. Given a rectangular shape, there are two ways to cross fold: (1) fold on the short edge on folding line 1 and then the long edge on folding line 2 (Figure 10); Figure 11 shows the crossfolded structure using method 1. (2) fold on the long edge first and then the short edge in Figure 12. Both methods were tested with a variation of thickness: 0.1, 0.3, 0.5, 1, 1.5, 2 mm. The size of 0.1 mm is the thinnest possible for manual handling after PolyJet printing. 

For this structure, it was created by the first fold along folding line 1, followed by folding along folding line 2. The bottom hinge was subsequently folded outwards. Materials with different glass transition temperatures (T_g_) were chosen and placed according to their movement. 

Opposite from method 1, method 2 was created by folding along fold line 2 across the structure, followed by folding along fold line 1. The bottom hinge was subsequently folded inwards.

**Figure 13:**
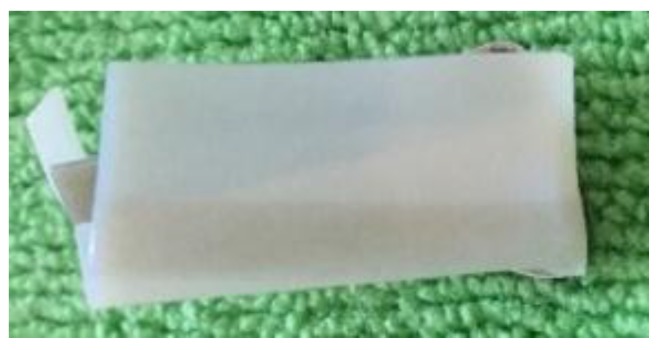
Crossfolding of final structure of method 2.

Figure 13 shows the crossfolded structure using method 2.

The results for design and crossfolding of multimaterial smart structures are described in Section 3. 

## 3. Results and Discussion

### 3.1. Shape Recovery of Single Material Specimens 

**Figure 14:**
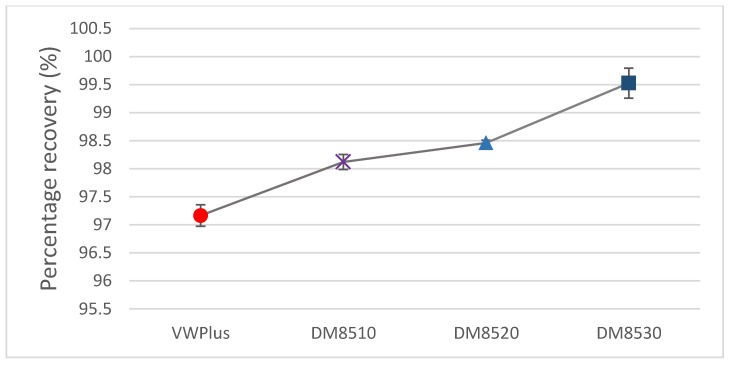
Percent recovery comparison of the four materials.

The recovery rates (in percentage) of four different materials are shown in Figure 14. DM8530 achieved the highest recovery rate whilst VeroWhitePlus had the lowest recovery rate. Due to the better recovery property, DM8530 should have a better ability to take the multi-folding process. 

### 3.2. Recovery of Crossfolded Single Material Smart Structures 

The programming procedure is as follows: set and maintain the water temperature at 70 °C. Put the specimen in the hot water for 2 min until the whole specimen becomes soft. Take out the specimen and fold vertically first, followed by folding horizontally. Then, put the folded specimen in the hot water again until it fully recovers. Repeat the programming stage and folding of the same specimen until it cracks and record the maximum folding times.

#### 3.2.1. Thickness of Folding Line 1 = 0.5 mm, Folding Line 2 = 1 mm

**Figure 15:**
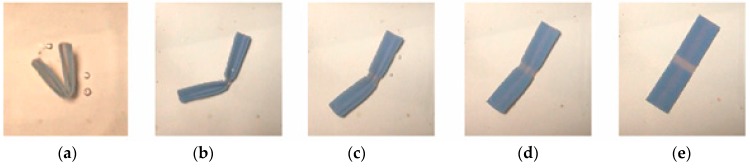
Method 1—the sequence of unfolding for 0.5 mm sample. (**a**) Unfold along folding line 1; (**b**) Continues to unfold; (**c**) Unfold along folding line 1 and unfold along folding line 2; (**d**) Continue to unfold along both folding lines; (**e**) Fully unfolded specimen.

**Figure 16:**
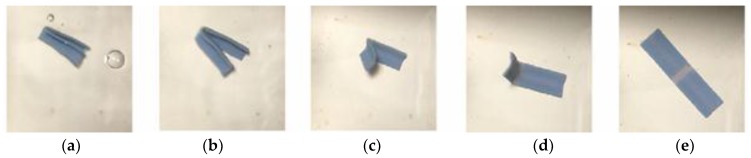
Method 2—the sequence of unfolding for 0.5 mm sample. (**a**) Unfold along folding line 1; (**b**) Continues to unfold; (**c**) Unfold along folding line 1 and unfold along folding line 2; (**d**) Continue to unfold along both folding lines; (**e**) Fully unfolded specimen.

As shown in Figure 15 and Figure 16aߝe, the sequence of the unfolding showed that 0.5 mm (folding line 1) opened earlier than 1.0 mm (folding line 2) due to the rapid response to thermal stimulus before the specimen was fully unfolded. The folding line 1 of 0.5 mm unfolded first in both cases, no matter how the folding sequence was done. This result was expected. The heat transfer was faster when the material was thinner, hence an earlier response. This result suggested that given the same unfolding sequence of folding line 1 and folding line 2, there were at least two ways to fold the structure into a compact form. In other words, crossfolding can increase the variety of smart structures. 

**Figure 17:**
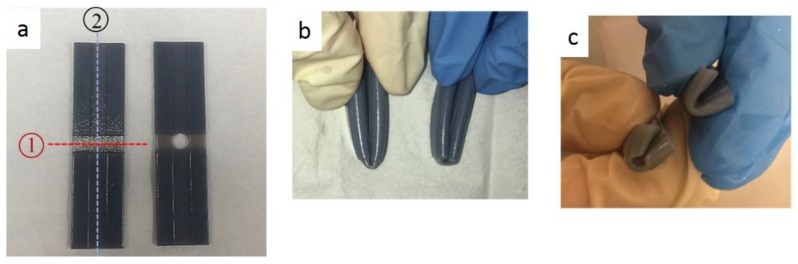
Crossfolding for the 0.5 mm sample. (**a**) Printed specimens with and without a hole; (**b**) Method 1—Folding line 2, followed by folding line 1; (**c**) Method 2—Folding line 1, followed by folding line 2.

Figure 17 shows that folding was easier when there was a hole in the center of the specimen, since the specimen with a hole eliminates the overlapping crossfolding region better than the specimen without a hole. It should be noted that the hole is the stress concentration region when it is subject to loading. However, in this cross-folding case, the hole is not subject to tensile load when folded. The hole replaces the stress concentrated region and hence leads to better cross-foldability. 

**Figure 18:**
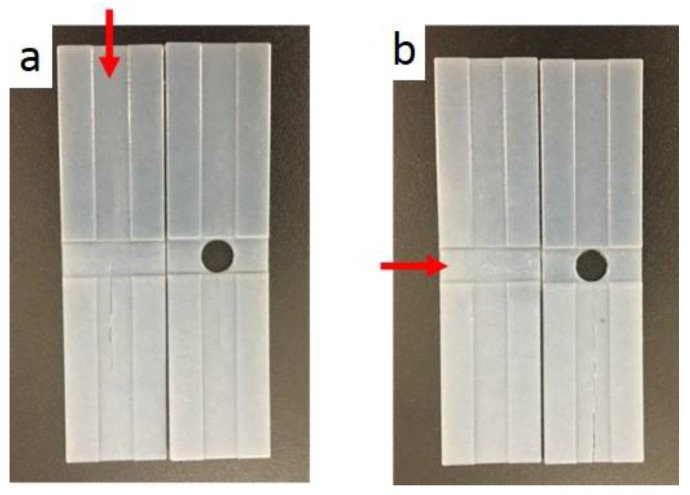
Fracture for the 0.5 mm sample. (**a**) After repeating method 1; (**b**) After repeating method 2.

As shown in Figure 18, a repeated test was performed for the same specimen and it started to break at the fourth time of programming. Similarly, it was observed that a hole in the center eliminated the overlapping crossfolding region and reduced cracks.

#### 3.2.2. Thickness of Folding Line 1 = 0.3 mm, Folding Line 2 = 1 mm 

**Figure 19:**
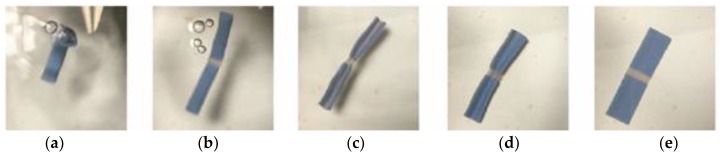
Method 1—the sequence of unfolding for the 0.3 mm sample from (**a**) Folded specimen was put in water bath and started to unfold; (**b**) Unfold along folding line 1; (**c**,**d**) Unfold along folding line 2; (**e**) Fully unfolded specimen.

**Figure 20:**
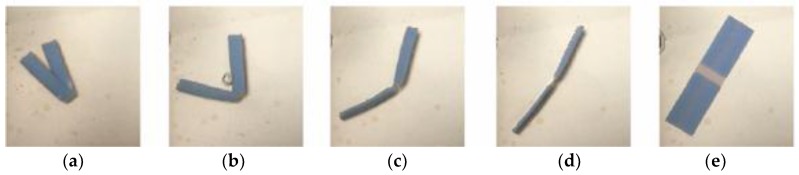
Method 2—the sequence of unfolding for the 0.3 mm sample from (**a**) Unfold along folding line 1; (**b**) Continue to unfold; (**c**,**d**) Unfolding along folding line 1 and unfold along folding line 2; (**e**) Fully unfolded specimne.

**Figure 21:**
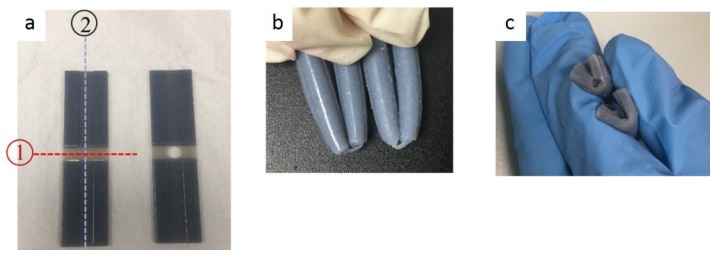
Crossfolding for the 0.3 mm sample for (**a**) Printed specimens with and without a hole; (**b**) Method 1—Folding line 2, followed by folding line 1; (**c**) Method 2—Folding line 1, followed by folding line 2.

**Figure 22:**
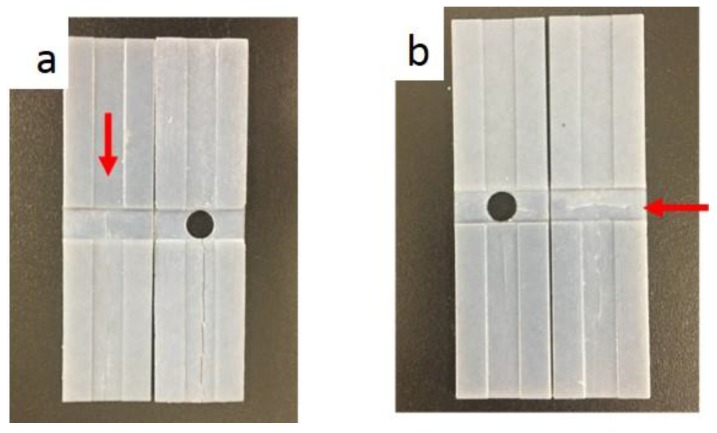
Fracture for the 0.3 mm sample (**a**) After repeating method 1; (**b**) After repeating method 2.

In Figure 19, Figure 20 and Figure 21, similar behaviors were observed except that in the repeated test, the specimen started to break at the second time of programming as shown in Figure 22. 

#### 3.2.3. Thickness of Folding Line 1 = 0.1 mm, Folding Line 2 = 1 mm 

**Figure 23:**
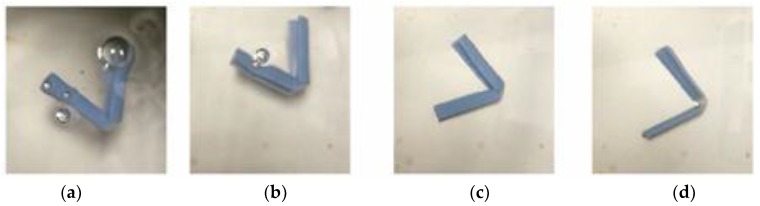
Method 2—the sequence of unfolding for the 0.1 mm sample. (**a**–**d**) Unfold along folding line 1.

**Figure 24:**
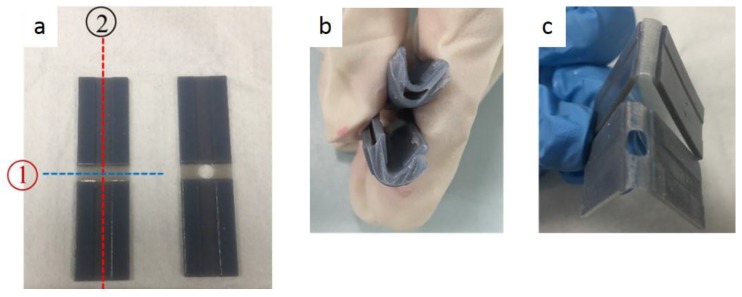
Crossfolding for the 0.1 mm sample. (**a**) Printed specimens with and without a hole; (**b**) Method 2—Folding line 1, followed by folding line 2; (**c**) After recovery.

**Figure 25:**
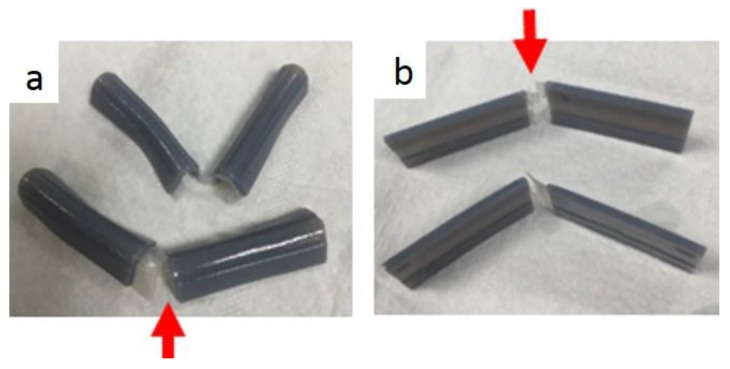
Fracture for the 0.1 mm sample. (**a**,**b**) Fracture at folding line 1.

Similar behaviors were observed as shown in Figure 23 and Figure 24, except that in the repeated test the specimen started to break at the first time of programming as shown in Figure 25.

The results of single material crossfolding are summarized in Table 3.

0.1 m, folding sequence 2 + 1 with hole and without, not recorded due to breakage.

### 3.3. Analysis of Tensile Test Results for Multimaterial Specimens

Crossfolding of the multimaterial structure is different from crossfolding of the single material structure, because material interfacial bonding in the multimaterial structure may play a role in shape setting. There is a concern that fractures or delamination may occur at the interface of different materials during shape setting. Therefore analysis of the mechanical properties of the multimaterial specimens is necessary. 

#### 3.3.1. Stress-Strain Curves

**Figure 26:**
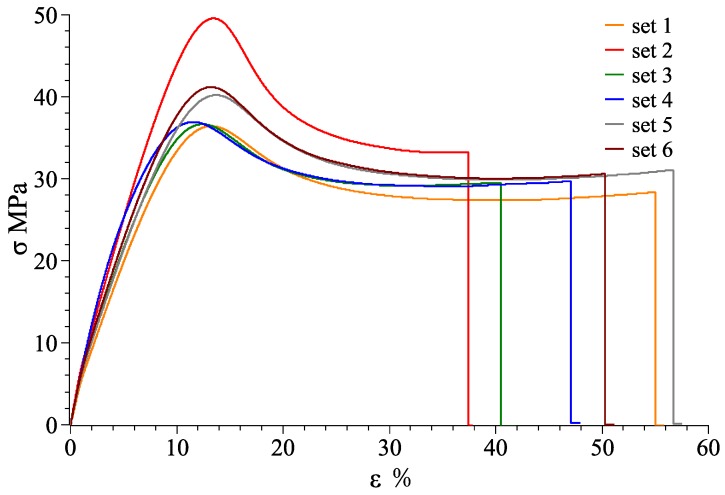
VeroWhitePlus, DM8510 stress–strain curve for the X-axis (6 specimens).

**Figure 27:**
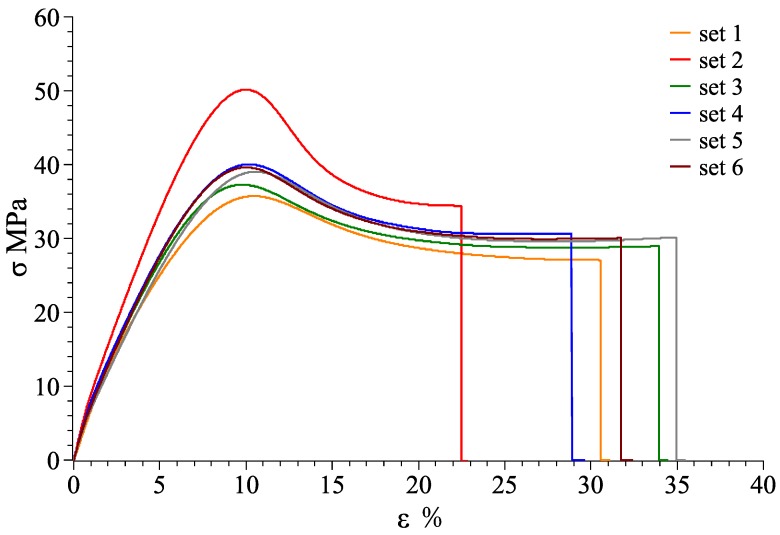
VeroWhitePlus, DM8510 stress–strain curve for the Y-axis (6 specimens).

**Figure 28:**
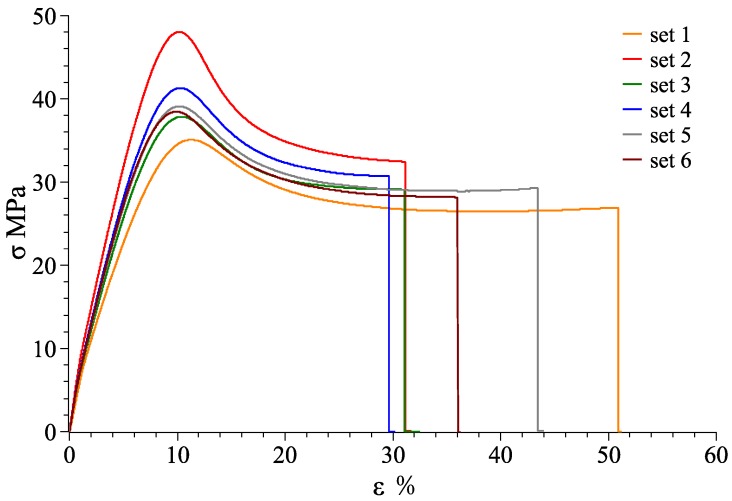
VeroWhitePlus, DM8510 stress–strain curve for the Z-axis (6 specimens).

At room temperature of 25 °C, stress-strain curves for material combination of VeroWhitePlus and D8510 are shown in Figure 26, Figure 27 and Figure 28 for the X, Y, Z axis. Other stress-strain curves and results are attached in Appendix A. For a combination of VeroWhitePlus and D8510, Figure 26 shows the stress-strain curves. The curves show the tensile results of six specimens of the same multi-material combination.

When two materials were combined symmetrically along the X-axis, the stress-strain curve was obtained as shown in Figure 26.

When two materials were combined symmetrically along the Y-axis, the stress-strain curve was obtained as shown in Figure 27.

When two materials were combined symmetrically along the Z-axis, the stress-strain curve was obtained as shown in Figure 28.

As observed, the red curve (#2) was likely an outlier as the curve deviated too much from the rest. After removing it, Table 4 shows the averages obtained from the remaining five curves.

#### 3.3.2. Theoretical Calculation versus Experimental Data

After calculations based on rule of mixture, the following results were obtained as shown in Table 5. For Young’s Modulus, all experimental values were lower than predicted values. One possible reason is the imperfect interfacial bonding that weakened the bond as discussed under single material investigation. Moreover, the rule of mixtures applies to composites embedded with long and continuous fibers. Our multi-material conditions are just an approximation of rule of mixtures situations as our fiber (material 1 or material 2) is not embedded in the composite. Materials were printed side by side, which resulted in less restraining force applied on the matrix from the fibers, hence making the specimen easier to deform (smaller Young’s Modulus). Additionally, the material in the experiments was not long enough to be classified as a long fiber. The general aspect ratio (length to diameter) of a long fiber should be at least 200 [30]. Moreover, Young’s Modulus of the X-axis was greater than Z-axis. According to the rule of mixtures, the X-axis and Z-axis should give the same Young’s Modulus. The reason could be that Z had a larger contact surface between the two different materials. When these two different materials were not 100% bonded, it allowed increased slippery movement and made the specimen more elastic (smaller Young’s Modulus). In addition, the direction of the bonding layer mattered. For the X-axis, the bonding direction was the same as the tensile test direction, whereas for the Y-axis, the bonding direction was perpendicular to the tensile test direction. As a result, the stronger part of the X-axis specimen took most of the elastic deformation. However, the stronger part of the Y-axis specimen was compressed by the weak part below it in the tensile test. Hence, different moduli were observed in the X-axis and Y-axis.

#### 3.3.3. Rupture Location and Interfacial Bonding Strength

**Figure 29:**
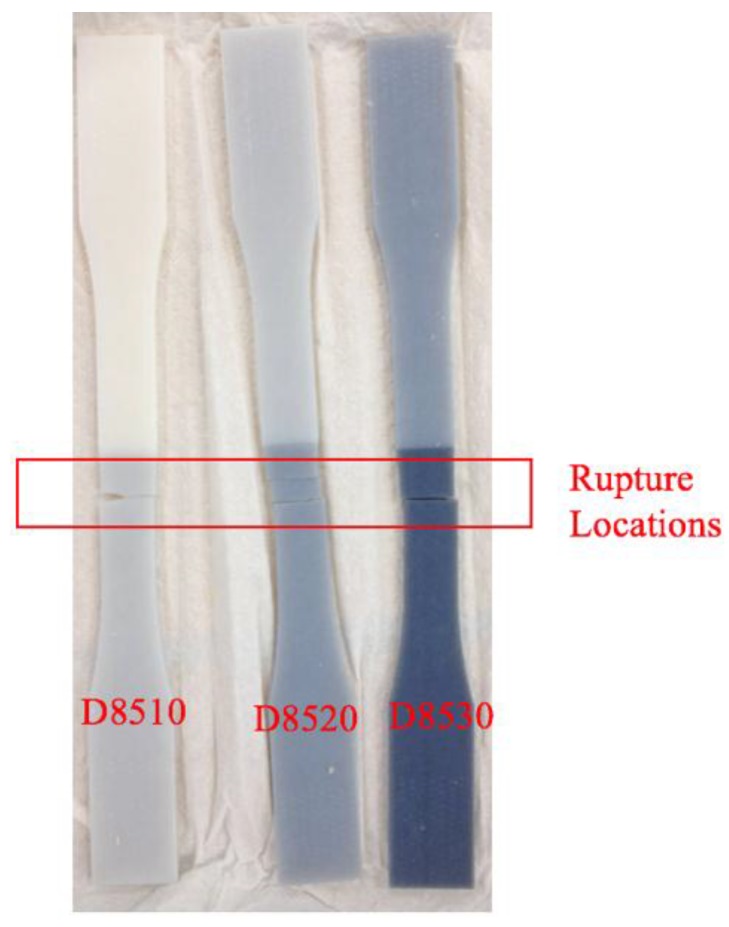
Rupture locations for the Y-axis.

For the Y-axis, the following ruptures were obtained (Figure 29). Young’s Modulus and Ultimate Tensile Stress decreased in the order: VeroWhitePlus > D8510 > D8520 > D8530. All three specimens did not break at the interface of the stronger and weaker materials but always at the weaker material. Since all specimens broke at the weaker material, not at the bonding, it meant that the interfacial bonding transmitted stress over to the weaker material. This observation corresponded to the test results and findings from Ge, Sakhaei, Lee et al. [8]. This result showed that the possibility of delamination or fracture at the interface of different materials during shape setting was negligible. It should be noted that the reference used for comparing bonding is different. When compared with weak and strong materials, the strength is ranked in the following order: weak material (rubber-like) < bonding < strong material (rigid-like). Hence all specimens broke at the weak material. However, if bonding is compared across different contact areas like Section 3.3.2, the larger the contact area, the poorer the bonding quality.

#### 3.3.4. Effect of the Material Combination Axis

**Figure 30:**
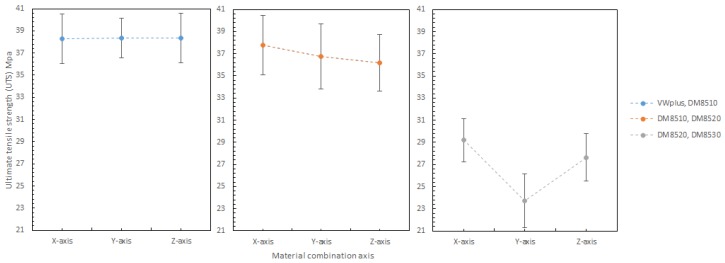
Combination axis effect on ultimate tensile strength (UTS) at 25 degree Celsius.

The material combination axis is the axis along which two materials are symmetrically combined. It is to be noted that the material combination axis can have a significant effect on maximum strain and Young’s modulus of all material combinations, but a mixed effect on Ultimate tensile strength (UTS). As shown in Figure 30, the material combinations suggested an inconsistent effect on UTS. The combination of DM8520 and DM8530 had lower UTS values. It might be related to the increased rubbery content in the materials, since the combination of DM8510 and DM8520 also showed slightly lower UTS values. The rubbery content in each material increased in the following order: VeroWhitePlus < DM8510 < DM8520 < DM8530.

**Figure 31:**
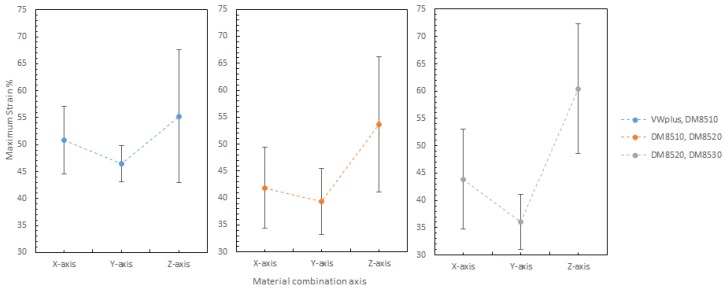
Combination axis effect on maximum strain 25 degree Celsius.

The material combination axis effect on maximum strain at 25 °C is shown in Figure 31. Clearly, the combination axis did affect the maximum strain. For all the different materials, the maximum strain increased in the order: Y-axis < X-axis < Z-axis. The reason was that the Z-axis had the most surface contact between two materials, allowing more slippery plastic deformation.

**Figure 32:**
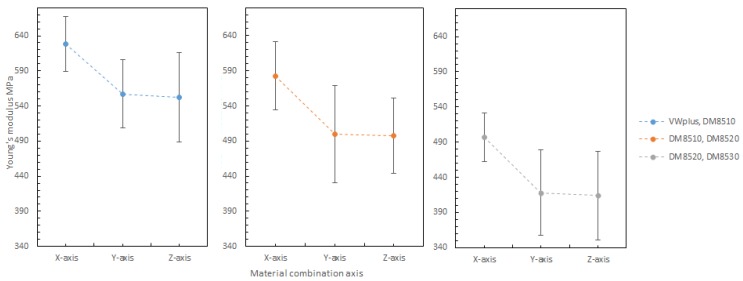
Combination axis effect on Young’s Modulus 25 degree Celsius.

Shown in Figure 32 are the material combination axis effects on Young’s Modulus, which were briefly mentioned in Table 5 earlier. For the Young’s Modulus, it decreases in the order: X-axis > Y-axis > Z-axis. The reason was the imperfect interfacial adhesion between the two materials. During 3D printing, the thermoset polymer’s properties give material irreversible cross-links after heating and curing. As 3D printing is line by line, there is a very high chance that the previous line is heated and half cured. When the next line is printed beside the first line, the bonding between these two lines is not 100% as cured materials cannot be bonded perfectly onto the previous layer. It is physical bonding between the two lines, instead of chemical bonding within the individual lines. This applies to different material boundary as well. Between material 1 and material 2, the first printed material would be cured after the second material was printed beside it, resulting in imperfect bonding at the material boundary which weakens the bonding and makes the specimen more elastic by allowing more slippery movement. Longitudinal adhesion along the loading direction was stronger than the transverse adhesion to the loading direction (see Section 3.3.2). This was the reason for the specimen with X-axis (longitudinal loading) orientation having a greater Young’s Modulus value than Y-axis (transverse loading).

### 3.4. Recovery of Crossfolded Multimaterial Smart Structures

**Figure 33:**
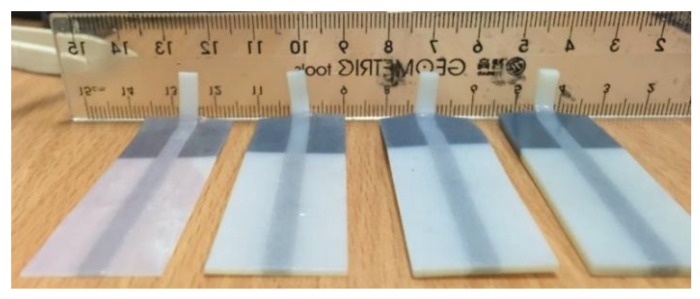
Folding line 3.

**Figure 34:**
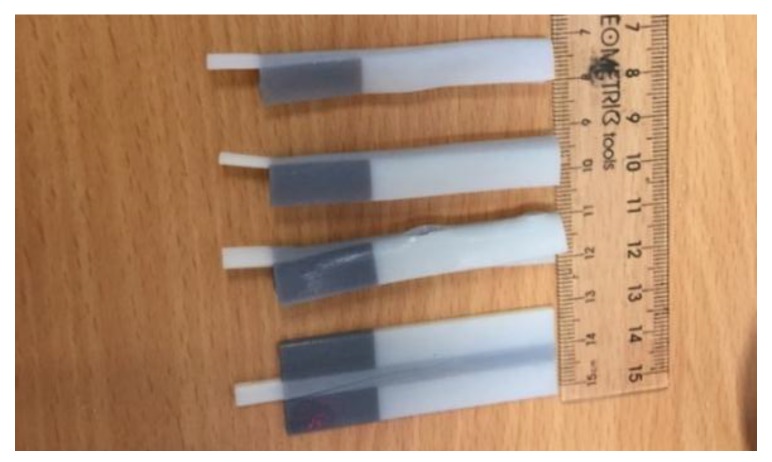
Folding line 2.

**Figure 35:**
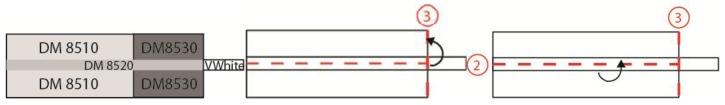
Materials and folding lines 2 and 3.

Figure 33 shows the folding line 3 of the petiole (stem) at the lower end of the petal, which involved just the bending of a single material into a bud shape. Figure 34 shows the folding line 2 of the petal which involved the folding of two different materials with different T_g_. Figure 35 shows the material placement and the axes of crossfolding of the structure. Fully folded structures are presented in Figure 11 and Figure 13 as shown earlier in Section 2.6.

Table 6 shows the variation of the different thicknesses of the structure. The upper boundary (maximum) thickness 0.5, 1.0, 1.5, 2.0 mm and lower boundary (minimum) thickness 0.1, 0.3 mm were printed. The different T_g_ materials were allocated to the structure. The structure represented the petal in a simplified geometric form instead of a curved shape for experimental purposes so that the observation of minor changes could be recorded accurately. However, if pressure was applied for folding line 3 or folding line 2, the structure could not be accurately controlled due to manual manipulation. The thickness of the structure was relevant when designing the flower with the combination of the basic designs [20]. From the experiment, it was observed that the thicker the folding line 3 on the structure, the longer it took for the structure to recover its original geometry. This was because a longer time was required for the center of a thicker sample to be heated to above its T_g_, which lengthened the overall recovery time. Similar to the folding line 3, it was observed that the thicker the folding structure, the longer it took for the structure to recover to its original geometry. The stem folded along folding line 1 had a constant thickness, therefore it was not included in Table 6.

The experiment showed that the thinner the thickness, the quicker the response time and the recovery rate for folding line 2 of the petal was longer than for the folding line 3 of the petiole. 

The multi-component strip of thickness 1 mm was 3D printed and programmed into the folded structure. Different materials were applied to the designed location on the strip to mimic the petal. Thermal stimulus was applied to activate the structure according to the material T_g_ and it was shown that for both methods, crossfolding was achievable in the experiments conducted. 

**Figure 36:**
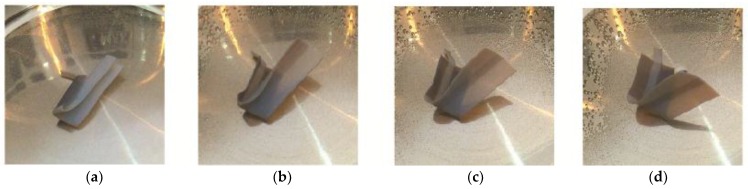

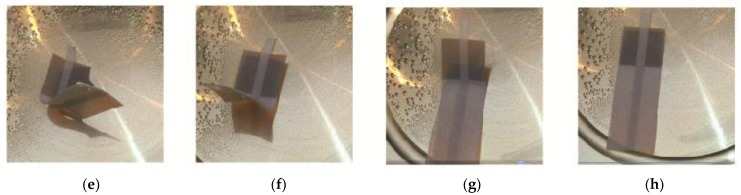
Method 1—the sequence of unfolding from (**a**) Folded specimen (**b**) DM8530 (dark grey) unfolding (**c**–**e**) Unfolding of DM8520 along folding line 2 (**f**) DM8510 unfolding (**g**) VeroWhitePlus unfolding (**h**) Specimen fully unfolded.

In Figure 36c–h, DM8530 had a lower T_g_ than DM8510. It can be seen that the sequence of the unfolding showed that DM8530 (darker grey) opened earlier than DM8510 (white material) with a higher T_g_ for the material to respond to thermal stimulus.

In method 2, folding line 2 first and then folding line 1, it was observed that a turning movement was created by the folding sequence. This depended on the folding order and material allocation on the strip. 

**Figure 37:**
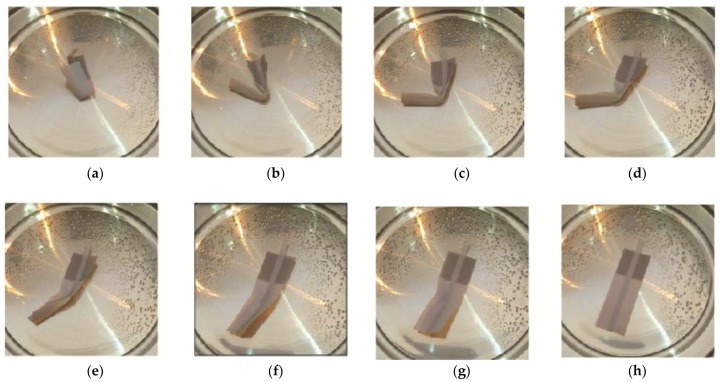
Method 2—the sequence of unfolding from (**a**) Folded specimen (**b**) DM8530 (dark grey) unfolding (**c**,**d**) DM8520 unfolding (**e**,**f**) DM8510 unfolding (**g**) VeroWhitePlus unfolding (**h**) Specimen fully unfolded.

This was similar to the cross folding method 1. It was due to the lower T_g_ of DM8530. It can be seen that the sequence of the unfolding at Figure 37d–h showed that the darker grey area (DM8530) opened earlier than the white material (DM8510).

## 4. Conclusions

For single material structures, a series of tests was conducted using specimens with different folding lines l and hinge thickness for the multi-folding process. The structures with smaller thicknesses recovered first independent of the folding sequence. Folding line l hinge of 0.5 mm thickness could only be folded three times, while 0.3 mm thickness could only be folded once prior to fracture cracks. The structure with 0.1 mm thickness had very low structural strength, as it was not able to recover back or even broke at the first folding. All specimens with a hole in the center eliminated the overlapping crossfolding region and reduced fracture cracks in the double folded region. For multimaterial structures, combining multimaterial components along different axes did affect the maximum strain and Young’s modulus of the composite, but the effect on UTS was mixed. A flower petal with sequential unfolding of multimaterial structures was designed. A combination of overlapping and non-overlapping folding lines was successfully demonstrated. Different thicknesses at different folding lines resulted in different recovery times. Overall, the development of design guidelines for accessing material cross-foldability was accomplished. Although crossfolding was possible in 4D printing, the radius of curvature during folding needs to be further minimized in the future. In future work, more precise predictions may be obtained by investigating 0.2 and 0.4 mm hinge thicknesses and observing the folding times before material failure. Finally, a complex origami structure will be investigated, which combines overlapping folding structures of single and multi-materials components. The potential applications of this preliminary research may include architectural kinetic façade and deployable space structures.

## Figures and Tables

**Table 1:** Material information.

Axis for Material Combination	Materials	Ratio	Printing Orientation
X-axis	VW, DM8510 DM8510, DM8520 DM8520, DM8530	50:50 50:50 50:50	Along X-axis
Y-axis	VW, DM8510 DM8510, DM8520 DM8520, DM8530	50:50 50:50 50:50	Along X-axis
Z-axis	VW, DM8510 DM8510, DM8520 DM8520, DM8530	50:50 50:50 50:50	Along X-axis

**Table 2:** Specimen material composition.

X-axis	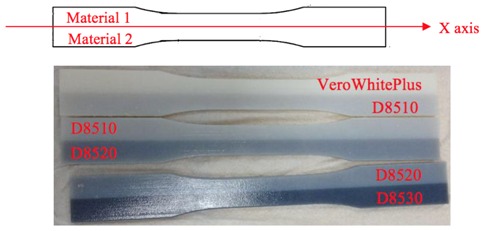
Y-axis	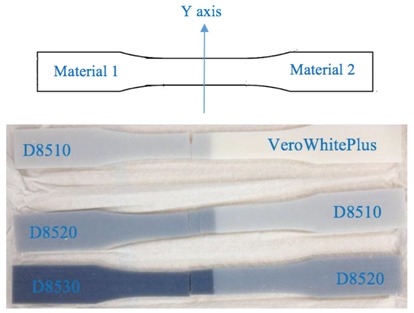
Z-axis	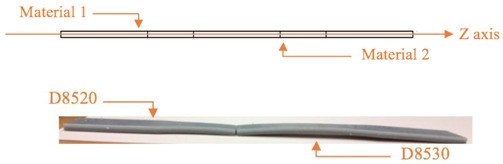

**Table 3:** Single material crossfolding.

Horizontal Hinge Thickness		Folding Sequence	Open Sequence	Maximum Folding Times	Crack with Visual Inspection	
0.5 mm	With hole	2 + 1	1 + 2	3	Less	1 = Folding line 1 2 = Folding line 2 Maximum folding times (Number of times cross-folded)
		1 + 2				
	Without hole	2 + 1			More	
		1 + 2				
0.3 mm	With hole	2 + 1	1 + 2	1	Less	
		1 + 2				
	Without hole	2 + 1			More	
		1 + 2				
0.1 mm	With hole	2 + 1	NA	NA	Break	
		1 + 2	1 + 2		Less	
	Without hole	2 + 1	NA		Break	
		1 + 2	1 + 2		More	

**Table 4:** VeroWhitePlus, DM8510 combination thermal-mechanical properties.

Parameter	X-Axis		Y-Axis		Z-Axis	
	Mean	Standard Deviation	Mean	Standard Deviation	Mean	Standard Deviation
Ultimate Tensile Stress (MPa)	38.273	±2.2	38.335	±1.8	38.354	±2.2
Maximum Strain (%)	50.795	±6.3	46.465	±3.4	55.247	±12.3
Young’s Modulus (MPa)	628.281	±38.8	557.266	±48.6	552.231	±63.9

**Table 5:** Young’s Modulus comparison for multi-material combination.

$E_{VW}$ **(MPa)**	$E_{DM8510}$ **(MPa)**	$V_{VW}$	$V_{DM8510}$	**Material Combination Axis**	$E_{Predicted}$ **(MPa)**	$E_{Experimental}$ **(MPa)**	**Error**
818.479	742.110	0.5		X	780.295	628.281 ± 38.8	19.5%
				Y	778.426	557.266 ± 48.6	28.4%
				Z	780.295	552.231 ± 63.9	29.2%
$E_{DM8510}$ **(MPa)**	$E_{DM8520}$ **(MPa)**	$V_{DM8510}$	$V_{DM8520}$	**Material Combination Axis**	$E_{Predicted}$ **(MPa)**	$E_{Experimental}$ **(MPa)**	**Error**
742.110	693.520	0.5		X	717.815	582.590 ± 48.5	18.8%
				Y	716.993	499.329 ± 69.3	30.4%
				Z	717.815	497.307 ± 53.6	30.7%
$E_{DM8520}$ **(MPa)**	$E_{DM8530}$ **(MPa)**	$V_{DM8520}$	$V_{DM8530}$	**Material Combination Axis**	$E_{Predicted}$ **(MPa)**	$E_{Experimental}$ **(MPa)**	**Error**
693.520	546.235	0.5		X	619.878	496.813 ± 35.0	19.9%
				Y	611.129	417.935 ± 60.8	31.6%
				Z	619.878	413.767 ± 63.5	33.3%

**Table 6:** Recovery time of folding line 3 and folding line 2.

Thickness (mm)	Time to Recover (s) (Temperature: 70−73 °C)	
	Folding Line 3	Folding Line 2
0.1	N/A	0.7
0.3	0.9	1.1
0.5	1.8	4
1	2	5
1.5	8	11
2	11	Breakage

**Figure A1:**
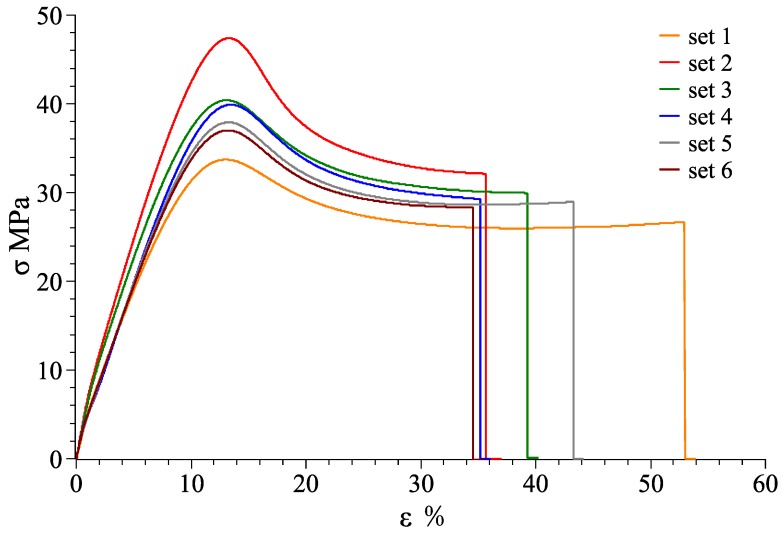
D8510, D8520 stress–strain curve for the X-axis (6 specimens).

**Figure A2:**
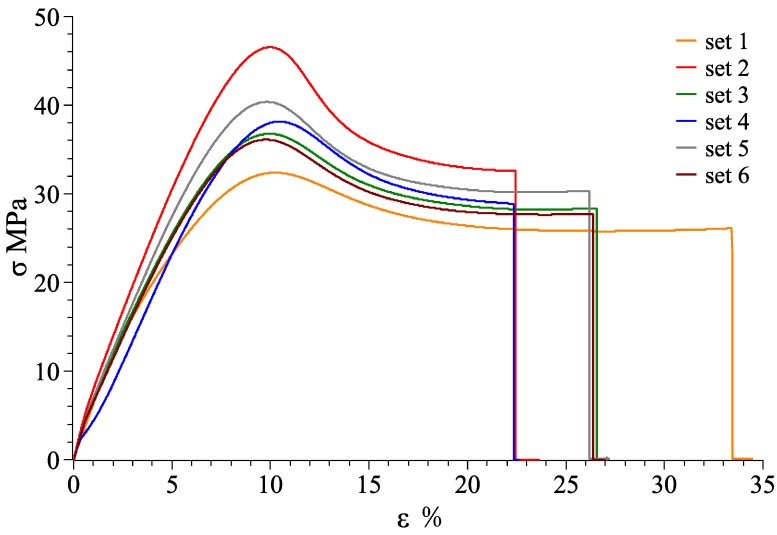
D8510, D8520 stress–strain curve for the Y-axis (6 specimens).

**Figure A3:**
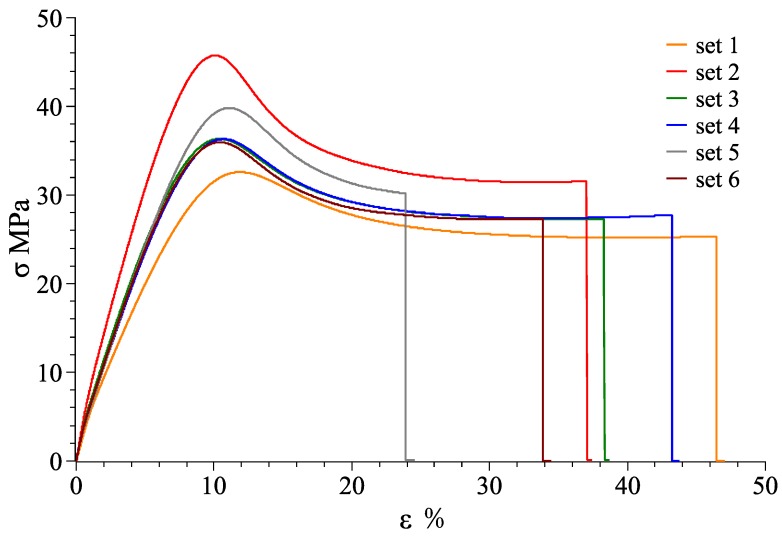
D8510, D8520 stress–strain curve for the Z-axis (6 specimens).

**Figure A4:**
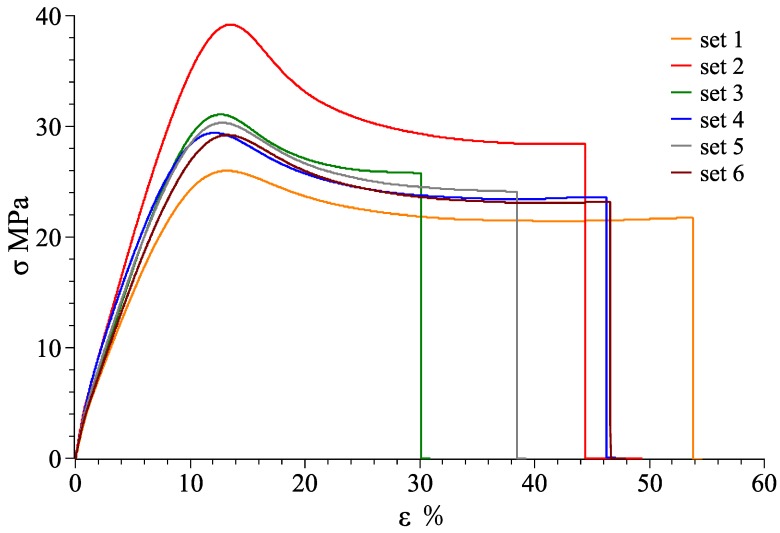
D8520, D8530 stress–strain curve for the X-axis (6 specimens).

**Figure A5:**
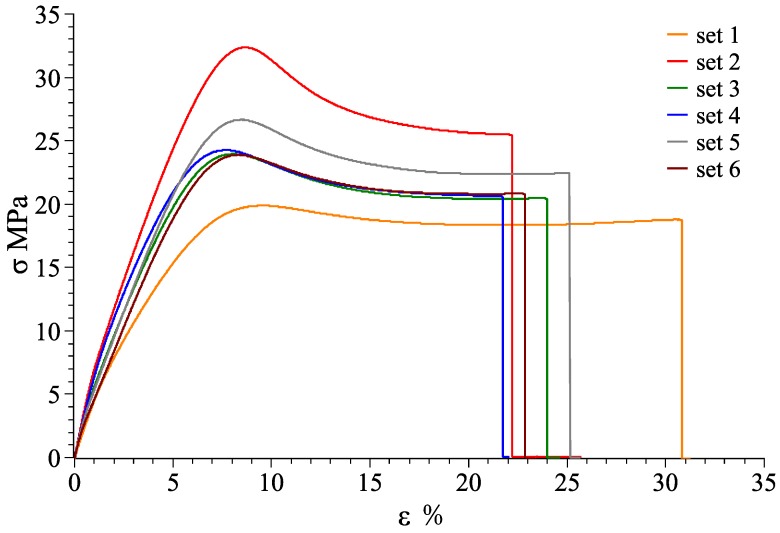
D8520, D8530 stress–strain curve for the Y-axis (6 specimens).

**Figure A6:**
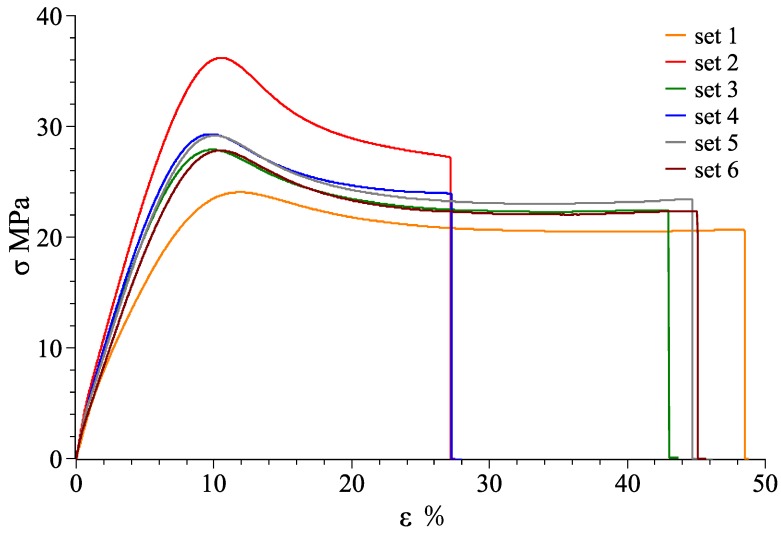
D8520, D8530 stress–strain curve for the Z-axis (6 specimens).

**Figure A7:**
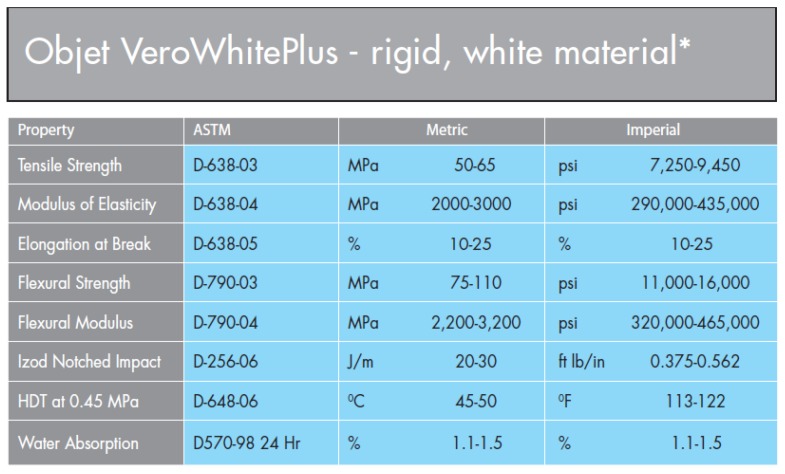
VeroWhitePlus material sheet from Stratasys. (Source: http://www.stratasys.com.cn/industries/education/research/4d%20printing%20project).

**Figure A8:**
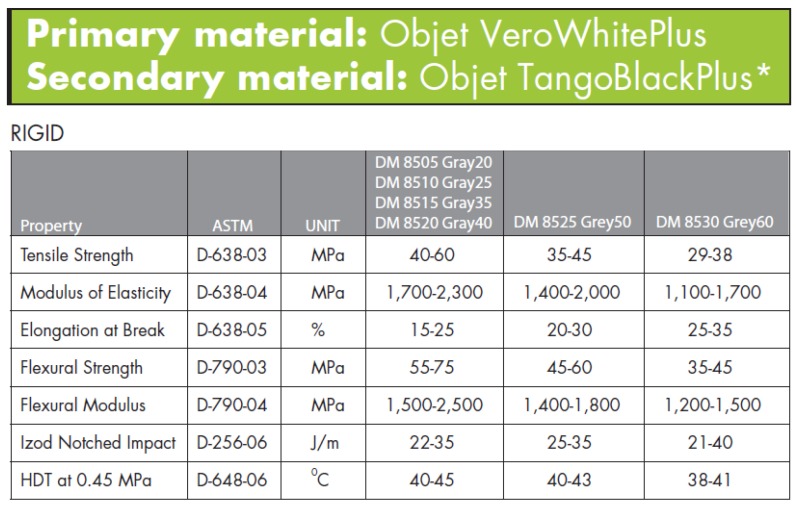
DM8510, DM8520, and DM8530 material sheet from Stratasys. (Source: http://www.stratasys.com.cn/industries/education/research/4d%20printing%20project. Measurement of shape recovery of single material specimens (raw data))

## Appendix A

**Multi-Material Stress–Strain Curves and Property Data**

For D8510, D8520:

X-axis:

Y-axis:

Z-axis:

For D8520, D8530:

X-axis:

Y-axis:

Z-axis:

**Table A1:** D8510, D8520 combination thermal-mechanical properties.

Parameter	X-Axis		Y-Axis		X-Axis	
	Mean	Standard Deviation	Mean	Standard Deviation	Mean	Standard Deviation
Ultimate Tensile Stress (MPa)	37.791	± 2.7	36.753	± 2.9	36.202	± 2.6
Maximum Strain (%)	41.910	± 7.5	39.341	± 6.1	53.617	± 12.5
Young’s Modulus (MPa)	582.590	± 48.5	499.329	± 69.3	497.307	± 53.6

set 2, abnormal data, removed from calculation.

**Table A2:** D8520, D8530 combination thermal-mechanical properties.

Parameter	X-Axis		Y-Axis		Z-Axis	
	Mean	Standard Deviation	Mean	Standard Deviation	Mean	Standard Deviation
Ultimate Tensile Stress (MPa)	29.202	± 1.9	23.727	± 2.4	27.650	± 2.1
Maximum Strain (%)	43.882	± 9.1	36.114	± 5.1	60.440	± 11.8
Young’s Modulus (MPa)	496.813	± 35.0	417.935	± 60.8	413.767	± 63.5

set 2, abnormal data, removed from calculation.

## Appendix B

VeroWhitePlus material sheet

## Appendix C

DM 8510, DM 8520 and DM 8530 material sheet

**Table A3:** VeroWhitePlus.

Sample No.	After Tension (mm)	Percent Elongation (%)	After Recovery (mm)	Percent Recovery (%)
1	28.06	10.7777	25.98	97.4339
2	28.00	10.5409	26.09	96.9996
3	28.12	11.0146	26.10	96.9601
4	28.25	11.5278	26.04	97.1970
5	28.20	11.3304	26.03	97.2365
Average	28.13	11.0383	26.05	97.1654

**Table A4:** DM8510.

Sample No.	After Tension (mm)	Percent Elongation (%)	After Recovery (mm)	Percent Recovery (%)
1	28.10	10.9356	25.75	98.3419
2	28.22	11.4094	25.82	98.0655
3	28.40	12.1200	25.83	98.0261
4	28.46	12.3569	25.83	98.0261
5	28.09	10.8962	25.80	98.1445
Average	28.25	11.5436	25.81	98.1208

**Table A5:** DM8520.

Sample No.	After Tension (mm)	Percent Elongation (%)	After Recovery (mm)	Percent Recovery (%)
1	28.36	11.9621	25.70	98.5393
2	28.29	11.6857	25.72	98.4603
3	28.20	11.3304	25.73	98.4208
4	28.15	11.1330	25.72	98.4603
5	28.35	11.9226	25.73	98.4208
Average	28.27	11.6068	25.72	98.4603

**Table A6:** DM8530.

Sample No.	After Tension (mm)	Percent Elongation (%)	After Recovery (mm)	Percent Recovery (%)
1	28.10	10.9356	25.34	99.9605
2	28.57	12.7912	25.43	99.6052
3	28.16	11.1725	25.48	99.4078
4	28.38	12.0411	25.50	99.3289
5	28.36	11.9621	25.50	99.3289
Average	28.31	11.7805	25.45	99.5263

**Table A7:** Glass transition temperatures of four materials used in the present research [20].

Material	Transition Temperature (T_g_)
VeroWhitePlus	55.6 °C
DM 8510	53.5 °C
DM 8520	51.6 °C
DM 8530	47.4 °C
